# Antioxidant Potential of a Wide Range of Commercial Fruit Powders and Grits for Food Applications

**DOI:** 10.1155/ijfo/8843447

**Published:** 2026-06-10

**Authors:** Iga Rybicka, Małgorzata Stanisz, Katarzyna Kupska, Joanna Pawlicka-Kaczorowska, Beata Zielińska, Włodzimierz Grajek, Anna Gliszczyńska-Świgło

**Affiliations:** ^1^ Department of Technology and Instrumental Analysis, Poznań University of Economics and Business, Poznań, Poland, put.poznan.pl; ^2^ Research and Development Department, Millano sp. z o.o. S.K.A, Poznań, Poland

**Keywords:** ABTS, antioxidant activity, DPPH, dried fruit, flavonoids, polyphenols

## Abstract

Dried fruits constitute a nutrient‐dense part of the everyday diet, providing different bioactive compounds in a convenient form. The aim of the study was to evaluate the antioxidant potential of a wide range of commercially available dried fruits. The analysis included the total phenolic content (TPC), total flavonoid content (TFC), total anthocyanin content (TAC), ability to scavenge DPPH^•^ radical (TEAC_DPPH_), and ABTS^•+^ radical cation (TEAC_ABTS_). The total of 60 products included 24 different fruits, divided into color‐based categories: black, red, and other fruits. Antioxidant capacity varied significantly among fruit groups, with, in general, black fruits showing the highest values; elderberry and honeyberry led in TPC, TFC, and TAC, while elderberry was superior in TEAC values. Among red fruits, rose hip had the highest TPC and the TEAC values, followed by strawberry. Fruits classified as other generally had lower antioxidant capacity, except citrus, which showed relatively high TPC and TFC. The results are useful for food producers engaged in product development and for consumers following a healthy diet, addressing current trends toward functional foods.

**Highlights**

• It is a handbook on the antioxidant capacity of 60 dried fruits for food applications.
• Polyphenols, flavonoids, anthocyanins, TEAC_ABTS_, and TEAC_DPPH_ were measured.
• Products were grouped into three color categories: black, red, and other fruits.
• Overall, the antioxidant potential was black fruits > > red fruits > > other fruits.
• The highest values were found for elderberry, rose hip, and honeyberry.

## 1. Introduction

Antioxidants can be produced by the human body or can be delivered from a diet including vegetables and fruits. They are necessary to reduce the harmful effects of excess free radicals, which can contribute to the development of various chronic diseases such as cardiovascular, neurodegenerative, carcinogenic, and immunological diseases [1]. Top antioxidants include vitamin C, vitamin E, selenium, zinc, and phytochemicals. Antioxidant phytochemicals are compounds naturally occurring in plants and include (poly)phenols (flavonoids, phenolic acids, lignans, and stilbens) and carotenoids [2]. They are found in a wide variety of foods, including fruits, vegetables, grains, spices, herbs, and fungi. Those recognized as the richest in antioxidants are berries, leafy greens such as spinach, dark chocolate, green tea, and nuts [3]. The antioxidant capacity of fruits contributes to their well‐documented health benefits, including reduced risk of cardiovascular disease, cancer, and neurodegenerative disorders. Fruits are rich in various nutrients and bioactive compounds—mostly polyphenols, carotenoids, vitamin C, and minerals [1, 2]. Berries (e.g., blueberry, strawberry, and raspberry), citrus fruits, grapes, and pomegranate are particularly high in these compounds. Chokeberry, blueberry, and strawberry are especially rich in anthocyanins–flavonoids responsible for their blue and red color, citrus fruits are a source of flavanones (e.g. hesperidin, narirutin, and naringin), while grapes have a high content of resveratrol [4, 5]. Therefore, it is suggested to consume a variety of fruits as generally different colors indicate the domination of different types of antioxidants.

Most of the food‐based dietary guidelines (FBDGs) suggest consuming about 400–600 g of fresh fruits and vegetables a day: starting from “at least 400 g” in the Czech Republic, Bulgaria, and Poland through “at least 500 g” in, for example, Latvia and Sweden to up to 600 g in Denmark and 500–800 g in Finland [6]. It is often translated into three to nine (mostly four to five) portions a day, where one portion corresponds to the size of a hand or 80–120 g of a fruit or vegetable. Moreover, most of the FBDGs clearly indicate that the vegetables should be chosen more often than fruits [6]. From the individual perspective, the best option is to consume fresh and regionally available fruits, as clearly stated in the Latvian recommendations. More detailed suggestions include the importance of different berries such as those presented for people living in the Baltic countries such as Denmark, Lithuania, Latvia, and Finland. Only some guidelines refer to the consumption of dried fruit, and mostly, they recommend limiting the consumption of dried fruits to “occasions” or “two to three portions a week,” where one portion is about 30 g. It mostly results from the higher calorie and sugar content in dried fruit compared to fresh fruit [6]. At the same time, it is underlined by nutritionists that dried fruits are more often an alternative to unhealthy snacks than to fresh fruits [7].

The increasing demand for convenient and nutritious snacks is fostering interest in dried fruits as valuable alternatives to traditional sweets [8]. Moreover, from the industrial perspective, fresh fruit is a perishable commodity due to its high moisture content. Drying inhibits the growth of undesirable microorganisms and enzymatic browning, while preserving the sensory and nutritional quality of the original raw material. Dried fruits, in the form of, for example, powder, or grits, are widely used in the confectionery, baking, and distilling industries to prepare various puddings, sauces, soft and alcoholic drinks, fruit‐flavored teas, chocolate, sweet snacks, and so forth. They are used as functional food additives to improve the nutritional value of foodstuffs, as flavorings or natural colorants (e.g., in ice cream, yogurts, and fruit bars) [9]. In 2023, the whole global dry fruit market was valued at $6.71 billion and is expected to expand to $10.41 billion by 2032, reflecting a compound annual growth rate (CAGR) of 5.2% over the forecast period [10]. The majority of global dried fruit production consists of dried grapes (commonly known as raisins) and table dates. The leading countries in dried fruit production worldwide in 2023–2024 were the United States (12% of market share), Turkey (11%), Saudi Arabia (10%), and Iran (10%) [11]. However, the market leader was Europe with a share of 28.3%. The largest producers of dried fruit in Europe are usually countries that are also leading producers of fresh fruits, such as Spain, Italy, and Poland. Poland plays a key role in the production of soft fruits in the European Union—it is the most important producer of cherries, raspberries, currants, and gooseberries, as well as one of the leaders in the export of these fruits [12].

Together with a growing trend to use ingredients with natural bioactive compounds, including phenolic compounds, the manufacturers can meet consumer demand for more nutritious choices. At the same time, consumers derive benefits from the bioactive properties and health‐promoting effects of these additives. Considering the significance of dried fruits from the individual, societal, and industrial perspectives, the aim of the study was to develop a comprehensive description of the antioxidant capacity, measured as the total phenolic content (TPC), total flavonoid content (TFC), total anthocyanin content (TAC), ability to scavenge DPPH^•^ radical (TEAC_DPPH_), and ABTS^•+^ radical cation (TEAC_ABTS_), of various dried fruits available on the European market. The list of 60 products includes 24 different fruits, divided into three color‐based categories. All samples are described in detail by providing a name, form, manufacturer, and photography, so they can be easily found on the market and used in a specific food product. Therefore, this paper provides a handbook on the total amount of phenolic antioxidants in a range of fruit components that can be used both by the food industry and households as food additives that increase the health value of the product and the daily diet. To the best of our knowledge, there are no studies covering such a wide range of fruit extracts available on the market.

## 2. Material and Methods

### 2.1. Material

The study was conducted for 60 commercial fruit components purchased on the Polish market in 2025. All samples were in the form of powder (40 samples) or grits (20 samples) and did not contain any additives such as oil or preservatives. Due to the varying number of products corresponding to a specific fruit, they were divided into three categories, namely, black fruits, red fruits, and other, based on the color of the raw fruit instead of depending on the type of fruit. A group of black fruits included honeyberry, bilberry, blueberry, blackcurrant, blackberry, chokeberry, and elderberry. Red fruits included redcurrant, raspberry, cranberry, hawthorn, rose hip, and strawberry, while other fruits included lemon, orange, grapefruit, bergamot, tangerine, date, pear, pineapple, peach, apricot, and mango. Tables 1, 2, and 3 provide basic characteristics of each sample including name and form, while Table S1 shows name, form, Latin systematic name, producer, and photography. As all samples were obtained as commercial products, detailed information on agricultural practices, processing, or storage conditions was not available.

**Table 1:** The total phenolic content (TPC), total flavonoid content (TFC), total anthocyanin content (TAC), Trolox equivalent antioxidant capacity with ABTS^•+^ (TEAC_ABTS_), and Trolox equivalent antioxidant capacity with DPPH^•^ (TEAC_DPPH_) of black fruits.

	Product (fruit)	Form	TPC mg GAE/g	TFC mg CE/g	TAC mg CGE/g	TEAC_ABTS_ *μ*mol Tx/g	TEAC_DPPH_ *μ*mol Tx/g
1.	Honeyberry	Powder	24.5 ± 1.7	11.3 ± 1.0	7.7 ± 0.03	185.4 ± 6.9	194.5 ± 14.0
2.	Honeyberry	Powder	23.0 ± 1.1	8.4 ± 0.2	17.4 ± 0.7	162.2 ± 18.4	174.3 ± 6.8
3.	Bilberry	Powder	21.7 ± 1.3	6.9 ± 0.2	10.1 ± 0.8	197.0 ± 10.9	163.8 ± 10.5
4.	Bilberry	Powder	12.5 ± 0.5	7.8 ± 0.3	2.4 ± 0.1	91.3 ± 3.7	96.5 ± 2.7
5.	Blueberry	Powder	20.3 ± 1.2	4.0 ± 0.2	4.1 ± 0.2	178.1 ± 3.8	167.4 ± 7.1
6.	Blueberry	Powder	19.7 ± 0.3	2.5 ± 0.2	6.0 ± 0.5	82.9 ± 2.9	79.6 ± 7.1
7.	Blueberry	Powder	19.6 ± 1.3	6.1 ± 0.3	2.7 ± 0.2	96.7 ± 2.6	98.8 ± 2.4
8.	Blackcurrant	Powder	15.5 ± 0.8	2.9 ± 0.1	0.3 ± 0.02	144.0 ± 10.4	90.4 ± 5.9
9.	Blackcurrant	Powder	20.0 ± 1.3	1.9 ± 0.1	10.1 ± 0.8	155.6 ± 2.0	132.6 ± 11.4
10.	Blackcurrant	Powder	12.1 ± 0.9	1.4 ± 0.1	0.8 ± 0.04	56.6 ± 6.8	109.4 ± 3.4
11.	Blackcurrant	Powder	21.3 ± 0.8	1.6 ± 0.1	5.4 ± 0.1	192.8 ± 10.0	194.0 ± 5.8
12.	Blackcurrant	Powder	28.7 ± 2.0	1.2 ± 0.1	9.2 ± 0.4	170.2 ± 2.0	146.9 ± 2.3
13.	Blackcurrant	Powder	13.7 ± 1.1	10.5 ± 0.2	2.8 ± 0.2	139.1 ± 5.7	121.0 ± 6.9
14.	Blackcurrant	Grits	11.3 ± 0.8	8.7 ± 0.3	2.8 ± 0.3	134.9 ± 3.7	104.4 ± 5.4
15.	Blackcurrant	Grits	18.0 ± 0.2	1.4 ± 0.1	1.5 ± 0.1	100.1 ± 7.5	121.5 ± 7.2
16.	Blackcurrant	Grits	11.3 ± 0.5	1.3 ± 0.1	0.4 ± 0.04	68.2 ± 6.9	90.5 ± 7.8
17.	Blackberry	Powder	19.0 ± 1.7	4.2 ± 0.2	1.5 ± 0.1	152.9 ± 9.6	176.1 ± 11.8
18.	Blackberry	Grits	14.1 ± 0.3	1.5 ± 0.1	2.6 ± 0.3	91.5 ± 5.2	110.8 ± 9.1
19.	Blackberry	Grits	9.9 ± 0.5	3.4 ± 0.2	0.5 ± 0.05	71.9 ± 6.3	75.5 ± 3.0
20.	Chokeberry	Powder	22.6 ± 0.3	4.0 ± 0.1	3.5 ± 0.2	199.7 ± 12.7	203.4 ± 5.0
21.	Chokeberry	Grits	15.0 ± 0.5	4.1 ± 0.3	2.4 ± 0.2	164.4 ± 8.1	171.7 ± 15.9
22.	Elderberry	Powder	10.5 ± 0.8	1.0 ± 0.1	1.0 ± 0.1	35.1 ± 0.3	33.0 ± 2.7
23.	Elderberry	Powder	43.4 ± 2.4	7.6 ± 0.6	1.4 ± 0.1	336.1 ± 15.4	355.6 ± 6.4
24.	Elderberry	Powder	41.6 ± 1.0	7.0 ± 0.3	32.3 ± 0.8	420.4 ± 10.6	398.8 ± 7.8

**Table 2:** The total phenolic content (TPC), total flavonoid content (TFC), total anthocyanin content (TAC), Trolox equivalent antioxidant capacity with ABTS^•+^ (TEAC_ABTS_), and Trolox equivalent antioxidant capacity with DPPH^•^ (TEAC_DPPH_) of red fruits.

	Product (fruit)	Form	TPC mg GAE/g	TFC mg CE/g	TAC mg CGE/g	TEAC_ABTS_ *μ*mol Tx/g	TEAC_DPPH_ *μ*mol Tx/g
25.	Redcurrant	Powder	9.7 ± 0.8	1.2 ± 0.1	0.1 ± 0.00	19.8 ± 0.8	24.9 ± 0.9
26.	Raspberry	Powder	9.4 ± 0.6	1.2 ± 0.1	8.9 ± 0.3	59.0 ± 1.2	77.0 ± 7.6
27.	Raspberry	Powder	10.8 ± 0.7	2.4 ± 0.1	1.7 ± 0.1	67.4 ± 2.8	92.3 ± 6.8
28.	Raspberry	Powder	10.6 ± 1.0	0.7 ± 0.02	0.3 ± 01	66.5 ± 3.9	98.3 ± 1.9
29.	Raspberry	Powder	10.6 ± 0.9	0.9 ± 0.1	3.1 ± 0.2	67.0 ± 3.3	89.6 ± 4.5
30.	Raspberry	Powder	13.3 ± 1.1	3.7 ± 0.2	0.4 ± 0.03	89.2 ± 6.9	93.9 ± 8.1
31.	Raspberry	Grits	8.4 ± 0.4	0.9 ± 0.1	2.1 ± 0.2	47.9 ± 3.2	64.5 ± 4.0
32.	Raspberry	Grits	12.8 ± 0.5	1.0 ± 0.1	0.6 ± 0.02	49.9 ± 3.6	59.5 ± 4.7
33.	Raspberry	Grits	7.2 ± 0.3	2.1 ± 0.1	0.8 ± 0.1	62.1 ± 1.2	59.4 ± 3.4
34.	Cranberry	Powder	12.2 ± 1.0	3.7 ± 0.1	3.1 ± 0.1	50.6 ± 3.3	68.8 ± 1.6
35.	Cranberry	Powder	9.0 ± 0.4	2.7 ± 0.2	0.2 ± 0.01	32.2 ± 1.9	30.4 ± 2.2
36.	Cranberry	Powder	10.8 ± 0.2	1.8 ± 0.1	5.4 ± 0.3	47.7 ± 1.4	80.2 ± 5.0
37.	Cranberry	Grits	13.2 ± 0.2	2.3 ± 0.1	4.3 ± 0.2	63.9 ± 4.6	75.8 ± 7.0
38.	Cranberry	Grits	3.5 ± 0.3	2.1 ± 0.2	1.5 ± 0.1	63.5 ± 5.9	66.8 ± 5.2
39.	Cranberry	Grits	12.9 ± 1.3	0.1 ± 0.00	0.7 ± 0.02	53.1 ± 2.8	69.5 ± 5.0
40.	Hawthorn	Powder	7.9 ± 0.6	3.1 ± 0.1	< 0.05	70.7 ± 9.7	70.6 ± 2.4
41.	Hawthorn	Grits	9.9 ± 0.4	2.1 ± 0.2	< 0.05	80.6 ± 2.3	56.8 ± 4.0
42.	Rose hip	Powder	25.4 ± 2.3	4.7 ± 0.3	n.d.	267.9 ± 24.3	219.6 ± 8.7
43.	Rose hip	Grits	22.0 ± 2.1	4.0 ± 0.2	n.d.	464.2 ± 57.8	229.4 ± 22.4
44.	Strawberry	Powder	20.1 ± 1.8	1.8 ± 0.1	1.5 ± 0.05	180.0 ± 10.7	162.2 ± 4.4
45.	Strawberry	Grits	15.0 ± 0.5	1.5 ± 0.1	0.5 ± 0.04	89.7 ± 6.0	92.1 ± 5.8

Abbreviation: n.d., not detected.

**Table 3:** The total phenolic content (TPC), total flavonoid content (TFC), total anthocyanin content (TAC), Trolox equivalent antioxidant capacity with ABTS^•+^ (TEAC_ABTS_), and Trolox equivalent antioxidant capacity with DPPH^•^ (TEAC_DPPH_) of other fruits.

	Product (fruit)	Form	TPC mg GAE/g	TFC mg CE/g	TAC mg CGE/g	TEAC_ABTS_ *μ*mol Tx/g	TEAC_DPPH_ *μ*mol Tx/g
46.	Lemon	Powder	3.2 ± 0.1	0.2 ± 0.01	n.d.	15.1 ± 1.5	11.6 ± 0.9
47.	Lemon	Powder	12.2 ± 0.7	1.6 ± 0.1	n.d.	37.0 ± 1.9	21.4 ± 1.4
48.	Lemon	Grits	39.6 ± 1.1	1.2 ± 0.1	n.d.	48.7 ± 4.5	13.6 ± 1.3
49.	Orange	Powder	12.5 ± 0.3	0.5 ± 0.04	n.d.	46.4 ± 3.5	15.2 ± 1.3
50.	Orange	Grits	12.2 ± 0.7	0.6 ± 0.1	n.d.	54.4 ± 1.4	15.3 ± 0.9
51.	Orange	Grits	11.4 ± 0.4	0.5 ± 0.03	n.d.	37.5 ± 1.3	15.2 ± 0.4
52.	Grapefruit	Grits	17.8 ± 1.0	0.5 ± 0.04	n.d.	39.4 ± 1.9	15.4 ± 0.7
53.	Bergamot	Grits	16.7 ± 1.5	2.2 ± 0.1	n.d.	49.8 ± 4.2	26.8 ± 1.6
54.	Tangerine	Powder	4.6 ± 0.2	0.1 ± 0.01	n.d.	5.3 ± 0.3	5.1 ± 0.1
55.	Date	Powder	4.9 ± 0.4	0.5 ± 0.03	n.d.	7.6 ± 1.0	13.7 ± 0.9
56.	Pear	Powder	5.0 ± 0.3	0.6 ± 0.04	n.d.	20.8 ± 1.5	20.7 ± 0.6
57.	Pineapple	Powder	3.2 ± 0.1	0.04 ± 0.00	n.d.	7.3 ± 0.6	11.5 ± 0.7
58.	Peach	Powder	3.6 ± 0.1	0.1 ± 0.00	n.d.	4.1 ± 0.3	7.9 ± 0.7
59.	Apricot	Powder	5.7 ± 0.5	0.2 ± 0.01	n.d.	10.6 ± 0.8	12.0 ± 0.1
60.	Mango	Powder	4.2 ± 0.4	0.04 ± 0.01	n.d.	22.0 ± 1.6	18.7 ± 0.3

Abbreviation: n.d., not detected.

### 2.2. Extraction Procedure

For the determination of TPC, TFC, TEAC_DPPH_, and TEAC_ABTS_ values, the extraction procedure was based on the water extraction described by Rybicka et al. [13]. The extraction of anthocyanins from the fruit samples was performed using acidified water to stabilize anthocyanins—0.1 mL of concentrated hydrochloric acid (Chempur, Piekary Śląskie, Poland) was mixed with 100 mL of demineralized water (Hydrolab System, Wiślina, Poland). In detail, 0.030 ± 0.005 g of each sample was weighed into an Eppendorf tube and mixed with 1.5 mL of acidified water. The mixture was shaken using a vortex mixer and sonicated for 30 min at controlled temperature (35^°^C ± 5^°^C) using an ultrasonic bath (ice added if necessary) (Sonorex Super RK 103 H, Bandelin electronic GmbH & Co., Berlin). Samples were centrifuged for 10 min at 14,500 rpm (MiniSpin plus, Eppendrof, Hamburg, Germany), and the supernatant was kept at −18°C until analysis. Three independent extracts were prepared for each sample.

### 2.3. TPC

The determination of TPC was performed according to the method of Singleton and Rossi [14]. Briefly, 0.02 mL of each extract was mixed with 0.2 mL of Folin–Ciocalteu (Analytichem, Zedelgem, Belgium) reagent in a spectrophotometric cuvette. After 3 min, 0.6 mL of 20% sodium carbonate (Chempur, Piekary Śląskie, Poland) and 3.18 mL of demineralized water were added. The sample was shaken and left in the dark at room temperature for 2 h. Then, the absorbance (*A*) was measured at 765 nm using a spectrophotometer (UV‐1280 spectrophotometer, Shimadzu Corporation, Kyoto, Japan). The results are expressed in gallic acid equivalents (mg GAE/g of product).

### 2.4. TFC

Determination of TFC was performed by the spectrophotometric method with silver chloride [15] as described in [13]. In brief, 0.05 mL of each extract was mixed in an Eppendorf tube with 1 mL of demineralized water and 0.06 mL of 5% NaNO_2_ (Chempur, Piekary Śląskie, Poland). After 5 min, 0.12 mL of 10% AgCl_3_ (Sigma‐Aldrich, Saint Louis, Missouri, United States) and 0.4 mL of 1 M NaOH (Chempur, Piekary Śląskie, Poland) were added. After the next 5 min, 0.37 mL of demineralized water was added. Before spectrophotometric measurement, the sample was centrifuged for 10 min at 14,500 rpm. The *A* of the supernatant was measured at 510 nm. The results were expressed in (±)‐catechin equivalents (mg CE/g of product).

### 2.5. TAC

The content of anthocyanins was determined using two buffer systems: potassium chloride (Chempur, Piekary Śląskie, Poland) buffer pH 1.0 and sodium acetate (Chempur, Piekary Śląskie, Poland) buffer pH 4.5 as described in Lako et al. [16]. Briefly, 0.2 mL of the extract was mixed with 1.8 mL of each buffer and measured against a blank (buffer with acidified water instead of an extract) at 510 and 700 nm using a spectrophotometer. The *A* was calculated using the formula:

A=A510700−ApH 1.04.5−A510700−ApH .

The total anthocyanin pigment concentration in the extract was calculated using the molar absorptivity of cyanidin‐3‐glucoside (26,900 dm^3^ mol^−1^ cm^−1^). The TAC was expressed in cyanidin‐3‐glucoside equivalents (mg CGE/g of product).

### 2.6. Determination of the Total Antioxidant Capacity With ABTS^•+^ Radical Cation (TEAC_ABTS_)

The total antioxidant capacity was determined using an assay with ABTS^•+^ radical cation according to Re et al. [17]. ABTS^•+^ radical cation was generated by a reaction of 0.0077 g of ABTS (BLD Pharmatech GmbH, Reinbek, Germany) dissolved in 1.8 mL of demineralized water with 0.2 mL of 0.0066 g/mL of potassium persulfate (Chempur, Piekary Śląskie, Poland). The reaction mixture was incubated in the dark at room temperature for 16 h. The ABTS^•+^ radical cation working solution was obtained by dilution with methanol (Th. Geyer GmbH & Co. KG, Renningen, Germany) to an *A* of 0.80 ± 0.02 at 734 nm. The *A* was measured 6 min after mixing 0.016 mL of the sample with 1.584 mL of the ABTS^•+^ working solution. The activity of fruits was expressed as the Trolox equivalent antioxidant capacity (TEAC_ABTS_) value (*μ*mol Tx/g of product). The standard curve of Trolox (TRC Toronto Research Chemicals, Toronto, Canada) was linear up to 24 mM. Additional dilutions of some extracts were needed when the TEAC_ABTS_ value measured was over the linear range of the standard curve.

### 2.7. Determination of the Total Antioxidant Capacity With DPPH^•^ Radical (TEAC_DPPH_)

The total antioxidant capacity of samples was determined using the DPPH method of Brand‐Williams et al. [18] with modifications described by Thaipong et al. [19]. In detail, the stock solution was prepared by dissolving 0.024 g DPPH^•^ (Sigma‐Aldrich, Saint Louis, Missouri, United States) in 100 mL of methanol and stored at −18°C until needed. The working solution was obtained by mixing 10 mL of stock solution with 45 mL of methanol to obtain an *A* of 1.10 ± 0.02 at 516 nm. Each extract (0.1 mL) was mixed with 1.9 mL of the DPPH^•^ working solution in the Eppendorf tube and allowed to react in the dark at room temperature for 2 h. The results were expressed as the TEAC_DPPH_ value (*μ*mol Tx/g of product). The standard curve of Trolox was linear up to 24 mM. Additional dilutions of some extracts were needed when the TEAC_DPPH_ value measured was over the linear range of the standard curve.

### 2.8. Statistical Analysis

The results are presented as mean ± standard deviation (SD) of triplicate determinations for each product. Mean values for three groups of fruits (black, red, and other) were compared using ANOVA with post hoc Tukey test (when data showed normal distribution and homoscedasticity) or nonparametric analysis with the Kruskal–Wallis test (when data did not show normal distribution). Normality was checked using the Shapiro–Wilk test, and homoscedasticity was checked using the Levene test, both at *α* = 0.05. Statistical analysis was carried out using Statistica 13.3 (2017) (Stat‐Soft, Inc., Tulsa, Oklahoma, United States).

## 3. Results and Discussion

Products were assigned to the color category (black, red, and other) based on the appearance of fresh fruit. Therefore, in some cases, the color of the tested product is different than the one resulting from the classification. For example, rose hip, red in the outer layer, is yellow‐orange after processing. This can be seen in the photographs presented in Table S1.

**Figure 1:**
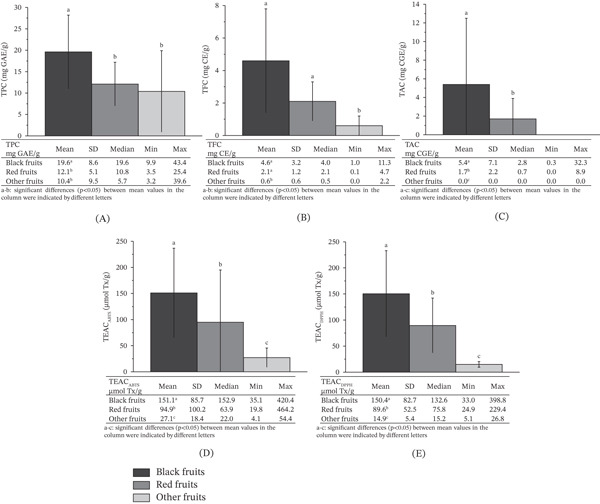
Comparison of the (A) total polyphenol content (TPC), (B) total flavonoid content (TFC), (C) total anthocyanin content (TAC), (D) Trolox equivalent antioxidant capacity with ABTS^•+^ (TEAC_ABTS_), and (E) Trolox equivalent antioxidant capacity with DPPH^•^ (TEAC_DPPH_) of black, red, and other fruits.

The antioxidant capacity of each dried fruit, expressed as TPC, TFC, TAC, TEAC_ABTS_, and TEAC_DPPH_ values, is presented in Table 1 (black fruits), Table 2 (red fruits), and Table 3 (other fruits). The tested products significantly differed in their antioxidant capacity (Figure 1). Black fruits (honeyberry, bilberry, blueberry, blackcurrant, blackberry, chokeberry, and elderberry) were characterized by the highest mean content of TPC, TFC, and TAC as well as the TEAC_ABTS_ and TEAC_DPPH_ values (19.6 mg GAE/g, 4.6 mg CE/g, 5.4 mg CGE/g, 151.1 *μ*mol Tx/g, and 150.4 *μ*mol Tx/g, respectively), followed by red fruits (redcurrant, raspberry, cranberry, hawthorn, rose hip, and strawberry: 12.1 mg GAE/g, 2.1 mg CE/g, 1.7 mg CGE/g, 94.9 *μ*mol Tx/g, and 89.6 *μ*mol Tx/g, respectively). Other fruits (lemon, orange, grapefruit, bergamot, tangerine, date, pear, pineapple, peach, apricot, and mango) had similar TPC (10.4 GAE/g) to red fruits, but much lower TFC, TEAC_ABTS_, and TEAC_DPPH_ values (0.6 mg CE/g, 27.1 *μ*mol Tx/g, and 14.9 *μ*mol Tx/g, respectively), and anthocyanins were not detected. Spectrophotometric methods for determining the antioxidant capacity of food are attractive due to their simplicity, low cost, and versatility. The results of spectrophotometric determination of phenolic compounds are usually higher than those obtained by high‐performance liquid chromatography, but a significant correlation between the two methods is reported (e.g., [20–22]). This means that qualitative and quantitative determination of individual phenolic compounds in well‐known and thoroughly tested plant material is not necessary for a quick estimate of the overall antioxidant capacity of a sample.

The fruits tested were in powder and grit forms, but only blackcurrant, raspberry, and cranberry were available in more than one product in both forms. For these fruits, no statistically significant differences were found in the parameters tested, except for the TEAC_DPPH_ value for raspberry. Raspberry powder had a statistically significant (*p* < 0.05) higher TEAC_DPPH_ value (90.2 *μ*mol Tx/g) than grits (61.2 *μ*mol Tx/g).

Due to the large range of results for individual fruits, no statistically significant differences were observed between mean values of fruits (all samples of one type of fruit) classified as black (*p* > 0.05). However, considering the average values of all parameters describing the antioxidant capacity, black fruits can be ordered as follows:

TPC: elderberry (mean content of 3 products = 31.8 mg GAE/g) > honeyberry (2 products, 23.7 mg GAE/g) > blueberry (3 products, 19.9 mg GAE/g) ≈ chokeberry (2 products, 18.8 mg GAE/g) ≈ bilberry (2 products, 17.1 mg GAE/g) ≈ blackcurrant (9 products, 16.9 mg GAE/g) > blackberry (3 products, 14.3 mg GAE/g).

TFC: honeyberry (9.8 mg CE/g) > bilberry (7.4 mg CE/g) > elderberry (5.2 mg CE/g) > blueberry (4.2 mg CE/g) ≈ chokeberry (4.0 mg CE/g) > blackcurrant (3.4 mg CE/g) ≈ blackberry (3.0 mg CE/g).

TAC: honeyberry (12.5 mg CGE/g) ≈ elderberry (11.6 mg CGE/g) > bilberry (6.2 mg CGE/g) > blueberry (4.3 mg CGE/g) > blackcurrant (3.7 mg CGE/g) > chokeberry (2.9 mg CGE/g) > blackberry (1.5 mg CGE/g).

TEAC_ABTS_: elderberry (263.9 *μ*mol Tx/g) > chokeberry (182.0 *μ*mol Tx/g) ≈ honeyberry (173.8 *μ*mol Tx/g) > bilberry (144.1 *μ*mol Tx/g) > blackcurrant (129.1 *μ*mol Tx/g) > blueberry (119.2 *μ*mol Tx/g) > blackberry (105.5 *μ*mol Tx/g).

TEAC_DPPH_: elderberry (262.5 *μ*mol Tx/g) > chokeberry (187.6 *μ*mol Tx/g) ≈ honeyberry (184.4 *μ*mol Tx/g) > bilberry (130.1 *μ*mol Tx/g) ≈ blackcurrant (123.4 *μ*mol Tx/g) ≈ blackberry (120.8 *μ*mol Tx/g) ≈ blueberry (115.5 *μ*mol Tx/g).

The comparison shows that elderberry or honeyberry is more valuable in TPC, TFC, and TAC, while in terms of the TEAC_ABTS_ and TEAC_DPPH_ values, elderberry is superior to other fruits in this group. It must also be mentioned that the antioxidant capacity of elderberry Sample No. 22 (Table 1) was much lower than that of other elderberry samples. According to the information available on the manufacturer’s website, the company used hot air drying, while, for example, Sample No. 24 was lyophilized. This could most likely explain its higher antioxidant capacity than Sample No. 22, as this method is considered more effective in preserving antioxidants than hot air drying [23].

In the group of red fruits, rose hip was characterized by statistically significantly higher (*p* < 0.05) TPC, TEAC_ABTS_, and TEAC_DPPH_ values than other fruits classified as red (one sample of redcurrant was excluded from the calculation). Statistically insignificant differences were observed between other fruits in this group (*p* > 0.05). Similarly to black fruits, considering the average values for all parameters describing the antioxidant capacity of each fruit, red fruits can be ordered as follows:

TPC: rose hip (2 products, 23.7 mg GAE/g) > strawberry (2 products, 17.6 mg GAE/g) > raspberry (8 products, 10.4 mg GAE/g) ≈ cranberry (6 products, 10.3 mg GAE/g) ≈ redcurrant (1 product, 9.7 mg GAE/g) ≈ hawthorn (2 products, 8.9 mg GAE/g).

TFC: rose hip (4.3 mg CE/g) > hawthorn (2.6 mg CE/g) > cranberry (2.1 mg CE/g) > strawberry (1.6 mg CE/g) ≈ raspberry (1.6 mg CE/g) > redcurrant (1.2 mg CE/g).

TAC: cranberry (2.5 mg CGE/g) > raspberry (2.2 mg CGE/g) > strawberry (1.0 mg CGE/g) > redcurrant (0.1 mg CGE/g) > hawthorn (<0.05 mg CGE/g) > rose hip (n.d.).

TEAC_ABTS_: rose hip (366.1 *μ*mol Tx/g) > strawberry (134.8 *μ*mol Tx/g) > hawthorn (75.6 *μ*mol Tx/g) > raspberry (63.6 *μ*mol Tx/g) > cranberry (51.8 mg GAE/g) > redcurrant (19.8 mg GAE/g).

TEAC_DPPH_: rose hip (224.5 *μ*mol Tx/g) > strawberry (127.1 *μ*mol Tx/g) > raspberry (79.3 mol Tx/g) > cranberry (65.3 *μ*mol Tx/g) ≈ hawthorn (63.7 *μ*mol Tx/g) > redcurrant (24.9 *μ*mol Tx/g).

The third group of products included lemon, orange, grapefruit, bergamot, tangerine, dates, pear, pineapple, peach, apricot, and mango. The antioxidant capacity of citrus fruits was generally much higher than other fruits from the third group. Their TPC, TFC, TEAC_ABTS_, and TEAC_DPPH_ values, with exception of tangerine and lemon No. 46 (Table 3), were in the range of 11.4–39.6 mg GAE/g, 0.5–2.2 CE/g, 37.0–54.4 *μ*mol Tx/g, and 13.6–26.8 *μ*mol Tx/g, respectively, whereas for other fruits, it was 3.2–5.7 mg GAE/g, 0.04–0.60 CE/g, 4.1–22.0 *μ*mol Tx/g, and 5.1–20.7 *μ*mol Tx/g, respectively. In detail:

TPC: lemon (3 products, 18.3 mg GAE/g) ≈ grapefruit (1 product, 17.8 mg GAE/g) ≈ bergamot (1 product, 16.7 mg GAE/g) > orange (3 products, 12.0 mg GAE/g) > apricot (1 product, 5.7 mg GAE/g) > pear (1 product, 5.0 mg GAE/g) ≈ date (1 product, 4.9 mg GAE/g) ≈ tangerine (1 product, 4.6 mg GAE/g) ≈ mango (1 product, 4.2 mg GAE/g) > peach (1 product, 3.6 mg GAE/g) ≈ pineapple (1 product, 3.2 mg GAE/g).

TFC: bergamot (2.2 mg CE/g) > lemon (1.0 mg CE/g) > pear (0.6 mg CE/g) ≈ grapefruit, orange and date (0.5 mg CE/g) > apricot (0.2 mg CE/g) > tangerine and peach (0.1 mg CE/g) > mango and pineapple (0.04 mg CE/g).

TAC: not detected.

TEAC_ABTS_: bergamot (49.8 *μ*mol Tx/g) ≈ orange (46.1 *μ*mol Tx/g) > grapefruit (39.4 *μ*mol Tx/g) > lemon (33.6 *μ*mol Tx/g) > mango (22.0 *μ*mol Tx/g) ≈ pear (20.8 *μ*mol Tx/g) > apricot (10.6 *μ*mol Tx/g) > date (7.6 *μ*mol Tx/g) ≈ pineapple (7.3 *μ*mol Tx/g) > tangerine (5.3 *μ*mol Tx/g) > peach (4.1 *μ*mol Tx/g).

TEAC_DPPH_: bergamot (26.8 *μ*mol Tx/g) > pear (20.7 *μ*mol Tx/g) ≈ mango (18.7 *μ*mol Tx/g) > lemon (15.6 *μ*mol Tx/g) ≈ grapefruit (15.4 *μ*mol Tx/g) ≈ orange (15.2 *μ*mol Tx/g) > date (13.7 *μ*mol Tx/g) > apricot (12.0 *μ*mol Tx/g) ≈ pineapple (11.5 *μ*mol Tx/g) > peach (7.9 *μ*mol Tx/g) > tangerine (5.1 *μ*mol Tx/g).

Although the antioxidant capacity of the fruits classified as “other” is much lower than that of black and red fruits (Figure 1; the exception is a comparable TPC in red fruits, Figure 1A), it is worth reaching for these fruits due to the different profile of polyphenolic compounds in these fruits than in berries [4, 5]. The main polyphenols in berry fruits described in the study are cyanidin, pelargonidin, peonidin, malvidin and delphinidin glycosides (anthocyanins), quercetin and kaempferol glycosides (flavonols), catechin, epicatechin (flavan‐3‐ols), gallic acid, proanthocyanidins, and ellagic acid and its conjugates, as well as chlorogenic acid derivatives. Anthocyanins constitute about 30%–70% of all phenolic compounds, depending on the berry [24, 25], so dried berries can serve as a natural dye or a source of natural colorants [26]. The main phenolic compounds in rose hip (fruit with the highest antioxidant capacity among fruits classified as red) are phenolic acids (gallic acid, ellagic acid, caffeic acid, and *p*‐coumaric acid), flavan‐3‐ols, and proanthocyanidins. Pear, apricot, and peach contain chlorogenic and neochlorogenic acids, quercetin and kaempferol glycosides, flavan‐3‐ols, and procyanidins [25]. Citrus fruits are a good source of flavanones, flavonols, and phenolic acids. Flavanones, hesperidin, and narirutin are dominant in oranges, hesperidin, and eriocitrin in lemons and limes, while naringin, narirutin, poncirin, and neohesperidin in grapefruits. These compounds influence the taste of citrus fruits as the rutinose moiety in hesperidin and narirutin contributes to a neutral taste, while the neohesperidose moiety in naringin and neohesperidin imparts a bitter flavor. Citrus flavonols include glycosides of kaempferol, apigenin, luteolin, quercetin, and diosmetin, and the main phenolic acids are free and bound hydroxycinnamic acids (ferulic, *p*‐coumaric, caffeic, and sinapic acid) and benzoic acids (vanillic, protocatechuic, gallic, and *p*‐hydroxybenzoic) [27].

The composition of fruits, and therefore their antioxidant capacity, is influenced by many factors, primarily the variety or cultivar of plant, location and cultivation conditions, ripeness stage, and storage conditions after harvesting. The antioxidant capacity of dried fruits can also be affected by drying method (e.g., [28]) or extraction procedure (e.g., [29]). Various solvents have been utilized for phenolics extraction (water and organic solvents, including acidified, or their mixtures) because there is no single solvent to dissolve the whole range of compounds [29]. Various researchers use different extraction solvents or conditions, or standards to express parameters describing the antioxidant capacity, which often refers to fresh fruits; thus, a direct comparison of results is difficult. Water is not the most effective solvent for phenolics, but it is a natural component of plant raw materials and is the best choice in terms of compatibility with most food products. Therefore, in this study, water‐soluble phenolics were used to evaluate the antioxidant potential of commercially available dried fruit powders and grits, so the comparison of the results obtained refers only to data on water‐soluble TPC (in GAE) or TAC (in CGE). For example, similar water‐soluble TPC was reported in rosehip (150.8–299.2 mg in 100 g d.w.) [30], blackberry (~1800–2300 mg in 100 g d.w.) [31], and apricot (0.65 g in 100 g d.w. [32]. Considering the average content of water in fruits (83%–90%), the results of TPC or TAC are generally typical for the fruits tested. Dragović‐Uzelac et al. [33] reported TPC in orange, apricot, peach, blueberry, and strawberry from the Croatian market at the levels of 1278, 506, 407, 2187, and 1127 mg GAE/kg f.w., respectively. Berry fruits (e.g., blackberry, raspberry, blackcurrant, and redcurrant) harvested in Italy contained from 0.21% to 0.83% of phenolics [34]. Berry fruits from Bosnia and Herzegovina provided from 4.6 to 199 mg of anthocyanins in 100 g of f.w. [35]. For strawberries, TPC and TAC were 79.3–164.1 mg/100 g f.w. and 10.7–24.9 mg/100 g f.w., respectively [36]. These results are consistent with the results of this study when expressed on a dry weight basis.

The trend to use ingredients with functional properties including antioxidant activity is growing in various branches of the food industry. For example, in the confectionery market, which accounted for $619 billion in 2025, one of the leading trends is the development of functional, healthy food products with natural ingredients [37]. Increased investment in research and development is projected to further accelerate this growth, with the market expected to grow annually by 5.47% (CAGR 2025–2030) [38]. The most valuable category of preserved pastry goods and cakes (about 40% of the market revenue) [37] is widely consumed in various regions of the world, but due to their composition (e.g., sugar or fat content), excessive consumption may contribute to the development of certain diet‐related diseases. Replacement of flour with dried fruit powders can considerably improve nutritional value, rheological, and organoleptic parameters, as well as microbiological and oxidative stability of the cakes [39]. Park and Chung [40] investigated the quality and antioxidant content of muffins containing chokeberry powder (0%, 10%, and 15%). Addition of this powder increased TPC and TFC, moisture content, hardness, and redness of the baked muffins but also decreased their volume. According to consumers, muffins with a 10% addition of chokeberry powder had acceptable sensory properties. In the study of Bełkowska et al. [41], commercial citrus fruit extract added to wafers in the amount of 0.13% (*w*/*w*) effectively delayed the oxidation processes of the lipid fraction during 10 months of storage at 18°C.

Chocolate products—the second most important category in the confectionery market, accounting for about 23% of market revenue [37]—are also shifting toward healthier options in response to growing consumer demand for better‐for‐you choices. This market is of particular importance for EU countries, which account for 65% of global chocolate exports, with the largest chocolate exporters being Germany, the Netherlands, and Poland [42]. The chocolate confectionery with fruit additives is present on the market for decades, but the trend to pay attention to its nutritional and health value has become noticeable in recent years. Only in 2024, two important Polish producers, Wedel and Wawel, independently launched the assortment of chocolate bars enriched with various fruits, nuts, and herbs clearly addressed to health‐conscious consumers. These were, for example, a milk chocolate bar with cherries and almonds and a date bar with cocoa and raspberries (Wedel) or “mini‐chocolates” with ginseng root, yerba mate, or ashwagandha extracts (Wawel). Considering the average annual consumption of 5.7 kg of chocolate per person in Poland [43], which corresponds to about 110 g of chocolate per week, and the data presented in the study, even a small addition of dried fruit to chocolate or chocolate filling, may contribute to increasing the antioxidant potential of the diet of people who consume chocolate products.

From a technological perspective, the literature suggests that dried fruit powders can be incorporated into chocolate matrices in small amounts (typically around 1% *w*/*w*) without compromising sensory quality or processing performance. At such levels, fruit powders act as functional enrichments rather than bulk ingredients, contributing polyphenols and antioxidant capacity while maintaining desirable texture, flavor, and appearance. For example, Żyżelewicz et al. [44] demonstrated that milk and dark chocolates enriched with 1% freeze‐dried powders from blueberries, raspberries, blackberries, or pomegranate pomace showed up to an 80% increase in phenolic content and higher antioxidant activity compared to control chocolates, while retaining good sensory acceptability. Similar effects have been reported for fruit extracts, where the addition of small amounts of acerola (*Malpighia glabra*) extract to dark chocolate significantly increased polyphenol content and antioxidant activity without adversely affecting rheological, textural, or sensory properties [45]. It is consistent with broader trends summarized in recent reviews, which indicate that fortification of chocolate with polyphenol‐rich plants or fruit ingredients is most effective at low inclusion levels (typically ≤ 1%–2% *w*/*w*), ensuring technological stability and consumer acceptance while enhancing functional properties [46].

It has to be underlined that dietary phenolics must be bioavailable to produce a specific biological effect; thus, the extent to which they are released from foods in the digestive tract, absorbed, and distributed in the human body must also be considered. Bioavailability of (poly)phenols varies considerably according to their chemical structure. It is also affected by the food matrix, the host health, enzyme‐, and microbial‐mediated biotransformation [47, 48]. Some (poly)phenols are absorbed in intact form, most exert their beneficial effect as metabolites; therefore, the data obtained by *in vitro* methods cannot be directly extrapolated to *in vivo* situation. However, the simultaneous application of different *in vitro* methods to evaluate antioxidant activity, as in the present study, offers a comprehensive perspective on the potential *in vivo* activity of food compounds. It is also clear that (poly)phenols can be bioavailable only if they are present in the consumed food or beverage [48]; thus, the knowledge about the *in vitro* antioxidant potential of fruit powders and grits, expressed as TPC, TFC, TAC, TEAC_ABTS_, and TEAC_DPPH_ values, may support the best choice of these products.

## 4. Conclusions

Significant differences in antioxidant capacity (TPC, TFC, TAC, TEAC_ABTS_, and TEAC_DPPH_) were observed among black, red, and other fruits, with black fruits showing the highest overall values. Among black fruits, elderberry and honeyberry exhibited the highest antioxidant potential, particularly in TPC, TFC, and TAC, with elderberry consistently superior in the TEAC values. Of the red fruits, rose hip had significantly higher TPC, TFC, and TEAC values than other red fruits, followed by strawberry. Anthocyanin levels varied, with cranberries and raspberries ranking highest in TAC within the red fruit group. Fruits in the category of “other” had much lower antioxidant capacity overall, except for citrus fruits, which showed relatively higher TPC and TFC compared to noncitrus. In general, fruit type and processing including drying method strongly influence antioxidant capacity, which should be considered when developing functional food products. Incorporation of dried fruit powders or grits into confectionery, especially chocolate and baked goods, can significantly enhance antioxidant potential while aligning with consumer trends toward healthier, functional foods. Given the numerous applications of dried fruits in food products, the data presented in this study provide important insights into the antioxidant activity of these ingredients.

## Author Contributions

Iga Rybicka: conceptualization, data curation, formal analysis, investigation, methodology, project administration, supervision, visualization, writing—original draft, and writing—review and editing. Małgorzata Stanisz: investigation. Katarzyna Kupska: investigation. Joanna Pawlicka‐Kaczorowska: investigation. Beata Zielińska: funding acquisition, project administration, and resources. Włodzimierz Grajek: funding acquisition, project administration, and resources. Anna Gliszczyńska‐Świgło: conceptualization, data curation, formal analysis, methodology, validation, visualization, writing—original draft, and writing—review and editing.

## Funding

The study was funded by the National Centre for Research and Development (FENG.01.01‐IP.01‐A029/23‐00).

## Ethics Statement

The authors have nothing to report.

## Conflicts of Interest

The authors declare no conflicts of interest.

## Supporting information


**Supporting Information** Additional supporting information can be found online in the Supporting Information section. Table S1: The characteristics of black, red, and other dried fruit (name, form, Latin name, producer, and additional information).

## Data Availability

The data that support the findings of this study are available from the corresponding author upon reasonable request.
