# Correction to “Antioxidant Potential of a Wide Range of Commercial Fruit Powders and Grits for Food Applications”

**DOI:** 10.1155/ijfo/9871461

**Published:** 2026-10-26

I. Rybicka, M. Stanisz, K. Kupska, J. Pawlicka‐Kaczorowska, B. Zielińska, W. Grajek, and A. Gliszczyńska‐Świgło, “Antioxidant Potential of a Wide Range of Commercial Fruit Powders and Grits for Food Applications,” *International Journal of Food Science*, 2026 (2026): 8843447, https://doi.org/10.1155/ijfo/8843447.

In the above‐mentioned article, the affiliation designation of the authors were listed incorrectly. Authors Małgorzata Stanisz, Katarzyna Kupska, Joanna Pawlicka‐Kaczorowska, Beata Zielińska, Włodzimierz Grajek, and Anna Gliszczyńska‐Świgło were listed at the incorrect affiliation. The correct affiliation list and author affiliations are shown below:

Iga Rybicka^1,2^ | Małgorzata Stanisz^2^ | Katarzyna Kupska^2^ | Joanna Pawlicka‐Kaczorowska^2^ | Beata Zielinska^2^ | Włodzimierz Grajek^2^ | Anna Gliszczynska‐Swigło^1^.

^1^Department of Technology and Instrumental Analysis, Poznan University of Economics and Business, Poznan, Poland | ^2^Research and Development Department, Millano sp. z o.o. S.K.A, Poznan, Poland.

This has been corrected in the online version accordingly.

We apologize for this error.

