# Supplementary material for: Antioxidant Potential of a Wide Range of Commercial Fruit Powders and Grits for Food Applications
Source: Int J Food Sci. 2026 Jun 10;2026:8843447. doi: 10.1155/ijfo/8843447 (PMC13250768; doi:10.1155/ijfo/8843447)
Supplement: Supplementary file 1 — Supporting Information Additional supporting information can be found online in the Supporting Information section. Table S1: The characteristics of black, red, and other dried fruit (name, form, Latin name, producer, and additional information). [file IJFO-2026-8843447-s001.zip › Supplementary Material_Table S1_final.docx]

**Table S1. The characteristics of black, red, and other dried fruit (name, form, latin name, producer, additional information)**

| **No.** | **Name** | **Form** | **Latin name** | **Producer (company, city, country)** | **Additional information^1^** | **Photography** |
| --- | --- | --- | --- | --- | --- | --- |
|  | **Black fruits** | | | | | |
| 1 | Honeyberry | powder | *Lonicera caerulea* | PPHU „AWB” Alina Becla, Handzlowka, Poland | 0-0.5 mm | 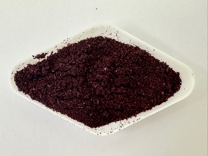 |
| 2 | Honeyberry | powder | *Lonicera caerulea* | Nutracevit Ltd, Lodz, Poland | 2 mm | 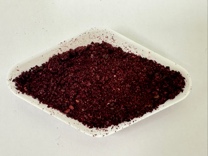 |
| 3 | Bilberry | powder | *Vaccinium myrtillus* | PPHU „AWB” Alina Becla, Handzlowka, Poland | dried | 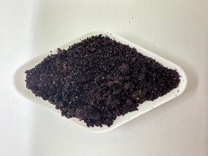 |
| 4 | Bilberry | powder | *Vaccinium myrtillus* | PAULA Ingredients Sp. z o o. Kalisz, Poland | dried | 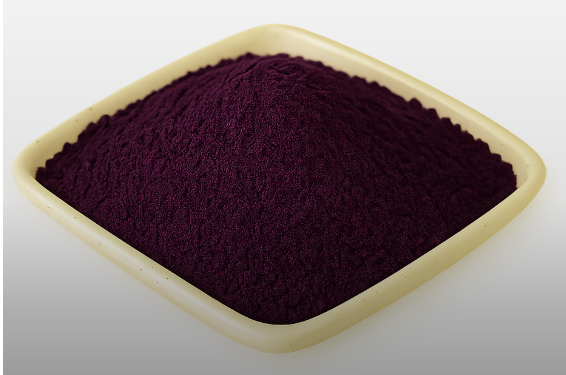 |
| 5 | Blueberry | powder | *Vaccinium corymbosum* | NATUREX, Avignon, France |  | 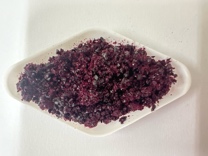 |
| 6 | Blueberry | powder | *Vaccinium corymbosum* | WPPH „ELENA” Żelazków, Poland | freeze-dried | 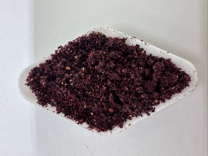 |
| 7 | Blueberry | powder | *Vaccinium corymbosum* | PAULA Ingredients Sp. z o o. Kalisz, Poland | dried,  0-1 mm | 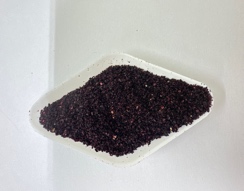 |
| 8 | Blackcurrant | powder | *Ribes nigrum* | PPHU „AWB” Alina Becla, Handzlowka, Poland | dried | 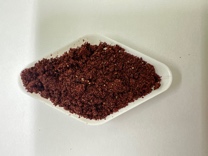 |
| 9 | Blackcurrant | powder | *Ribes nigrum* | Celiko S. A. Poznan, Poland | ground | 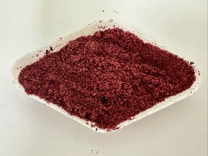 |
| 10 | Blackcurrant | powder | *Ribes nigrum.* | PPHU „AWB” Alina Becla, Handzlowka, Poland | ground | 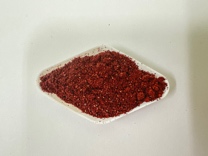 |
| 11 | Blackcurrant | powder | *Ribes nigrum* | NATUREX, Avignon, France |  | 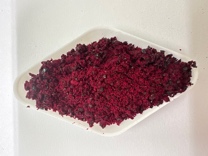 |
| 12 | Blackcurrant | powder | *Ribes nigrum* | WPPH „ELENA” Żelazków, Poland | freeze-dried | 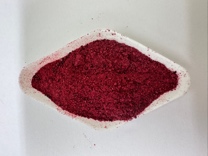 |
| 13 | Blackcurrant | powder | *Ribes nigrum.* | PAULA Ingredients Sp. z o o. Kalisz, Poland | 0-0.5 mm | 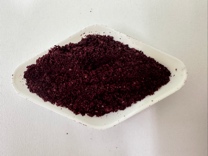 |
| 14 | Blackcurrant | grit | *Ribes nigrum.* | PAULA Ingredients Sp. z o o. Kalisz, Poland | 4-6 mm | 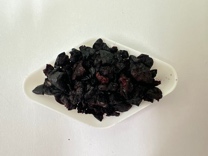 |
| 15 | Blackcurrant | grit | *Ribes nigrum.* | KACZMAREK KOMPONENTY, Poland | 2-5 mm | 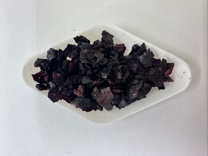 |
| 16 | Blackcurrant | grit | *Ribes nigrum* | PPHU „AWB” Alina Becla, Handzlowka, Poland |  | 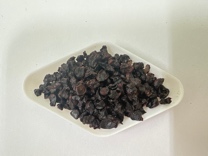 |
| 17 | Blackberry | powder | *Rubus fruticosus* | PPHU „AWB” Alina Becla, Handzlowka, Poland | dried | 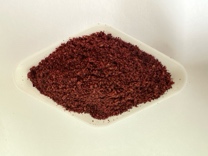 |
| 18 | Blackberry | grit | *Rubus fruticosus* | KACZMAREK KOMPONENTY, Poland | 2-5 mm | 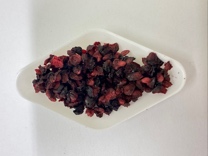 |
| 19 | Blackberry | grit | *Rubus fruticosus* | PAULA Ingredients Sp. z o o. Kalisz, Poland | 2-5 mm | 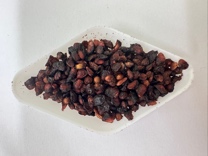 |
| 20 | Chokeberry | powder | *Aronia melanocarpa* | PPHU „AWB” Alina Becla, Handzlowka, Poland | ground | 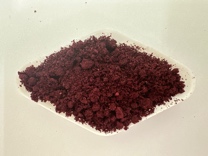 |
| 21 | Chokeberry | grit | *Aronia melanocarpa* | PPHU „AWB” Alina Becla, Handzlowka, Poland |  | 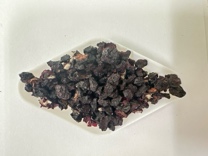 |
| 22 | Elderberry | powder | *Sambucus nigra* | PPHU „AWB” Alina Becla, Handzlowka, Poland |  | 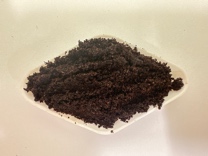 |
| 23 | Elderberry | powder | *Sambucus nigra* | NATUREX, Avignon, France |  | 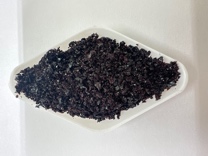 |
| 24 | Elderberry | powder | *Sambucus nigra* | WPPH „ELENA” Żelazków, Poland | freeze-dried | 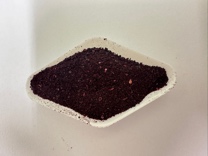 |
| **Redcurrant** | | | | | | |
| 25 | Redcurrant | powder | *Ribes nigrum* | PPHU „AWB” Alina Becla, Handzlowka, Poland | dried | 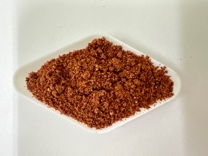 |
| 26 | Raspberry | powder | *Rubus idaeus* | Celiko S. A. Poznan, Poland |  | 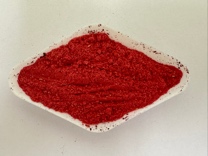 |
| 27 | Raspberry | powder | *Rubus idaeus* | PPHU „AWB” Alina Becla, Handzlowka, Poland | dried | 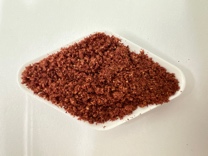 |
| 28 | Raspberry | powder | *Rubus idaeus* | NATUREX, Avignon, France |  | 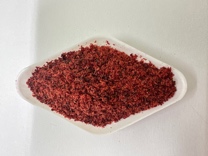 |
| 29 | Raspberry | powder | *Rubus idaeus* | WPPH „ELENA” Żelazków, Poland | freeze-dried | 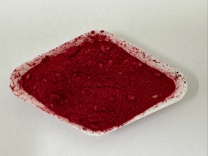 |
| 30 | Raspberry | powder | *Rubus idaeus* | PAULA Ingredients Sp. z o o. Kalisz, Poland | dried,  0-1 mm | 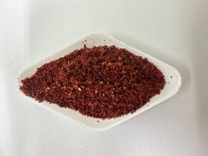 |
| 31 | Raspberry | grit | *Rubus idaeus* | WPPH „ELENA” Żelazków, Poland | freeze-dried, 0-6 mm | 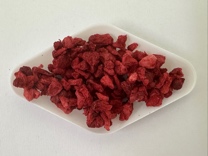 |
| 32 | Raspberry | grit | *Rubus idaeus* | KACZMAREK KOMPONENTY, Poland | 2-5 mm | 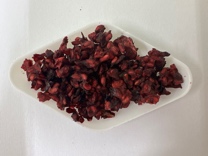 |
| 33 | Raspberry | grit | *Rubus idaeus* | PAULA Ingredients Sp. z o o. Kalisz, Poland | dried,  3-5 mm | 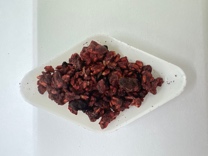 |
| 34 | Cranberry | powder | *Vaccinium macrocarpon* | Celiko S. A. Poznan, Poland |  | 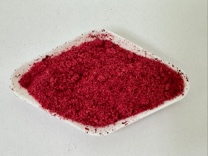 |
| 35 | Cranberry | powder | *Vaccinium macrocarpon* | PPHU „AWB” Alina Becla, Handzlowka, Poland | dried | 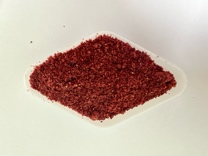 |
| 36 | Cranberry | powder | *Vaccinium macrocarpon* | WPPH „ELENA” Żelazków, Poland | freeze-dried | 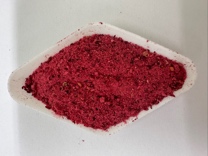 |
| 37 | Cranberry | grit | *Vaccinium macrocarpon* | WPPH „ELENA” Żelazków, Poland | freeze-dried, 0-6 mm | 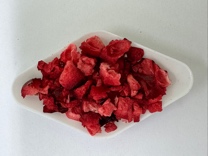 |
| 38 | Cranberry | grit | *Vaccinium macrocarpon* | KACZMAREK KOMPONENTY, Poland | 2-5 mm | 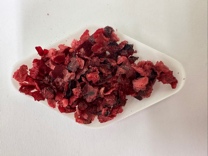 |
| 39 | Cranberry | grit | *Vaccinium macrocarpon* | PAULA Ingredients Sp. z o o. Kalisz, Poland | dried,  4-6 mm | 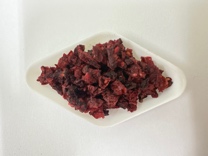 |
| 40 | Hawthron | powder | *Crataegus monogy* | PPHU „AWB” Alina Becla, Handzlowka, Poland | ground | 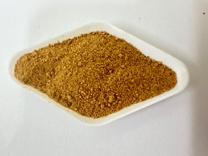 |
| 41 | Hawthron | grit | *Crataegus monogy* | PPHU „AWB” Alina Becla, Handzlowka, Poland |  | 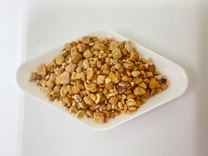 |
| 42 | Rose hip | powder | *Rosa canina* | PPHU „AWB” Alina Becla, Handzlowka, Poland | ground | 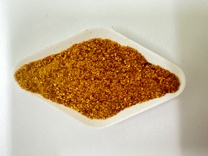 |
| 43 | Rose hip | grit | *Rosa canina* | PPHU „AWB” Alina Becla, Handzlowka, Poland |  | 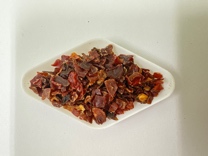 |
| 44 | Strawberry | powder | *Fragaria × ananassa* | NATUREX, Avignon, France |  | 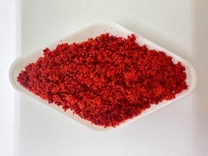 |
| 45 | Strawberry | grit | *Fragaria ananassa* | KACZMAREK KOMPONENTY, Poland | 2-5 mm | 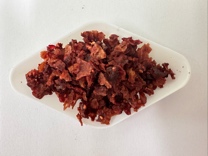 |
| **Other fruits** | | | | | | |
| 46 | Lemon | powder | *Citrus limon* | NATUREX, Avignon, France |  | 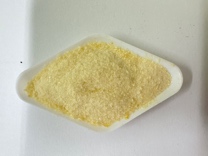 |
| 47 | Lemon | powder | *Citrus limon* | KACZMAREK KOMPONENTY, Poland | with peel 800 μm | 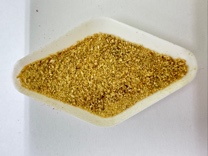 |
| 48 | Lemon | grit | *Citrus limon* | KACZMAREK KOMPONENTY, Poland | organic. 2-5 mm | 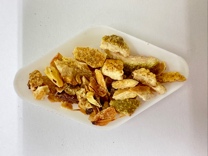 |
| 49 | Orange | powder | *Citrus sinensis* | KACZMAREK KOMPONENTY, Poland | with peel 800 μm | 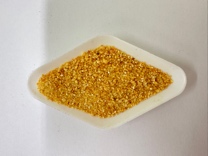 |
| 50 | Orange | grit | *Citrus sinensis* | KACZMAREK KOMPONENTY, Poland | organic,  with peel,  2-5 mm | 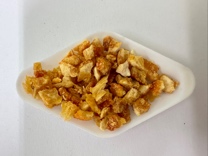 |
| 51 | Orange | grit | *Citrus sinensis* | KACZMAREK KOMPONENTY, Poland | 0.3-1.7 mm | 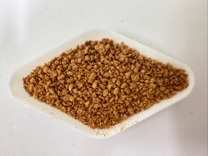 |
| 52 | Grapefruit | grit | *Citrus paradisi* | KACZMAREK KOMPONENTY, Poland | with peel,  0.6-2 mm | 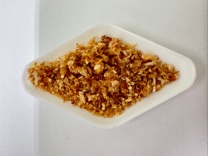 |
| 53 | Bergamot | grit | *Citrus bergamia* | KACZMAREK KOMPONENTY, Poland | 1-3 mm | 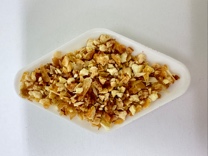 |
| 54 | Tangerine | powder | *Citrus reticulata* | NATUREX, Avignon, France |  | 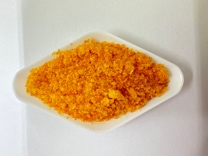 |
| 55 | Date | powder | *Phoenix dactylifera* | NATUREX, Avignon, France |  | 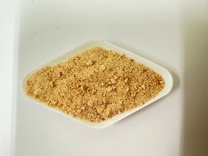 |
| 56 | Pear | powder | *Pyrus communis* | NATUREX, Avignon, France |  | 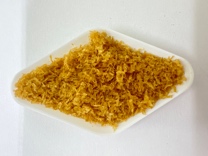 |
| 57 | Pineapple | powder | *Ananas comosus* | NATUREX, Avignon, France |  | 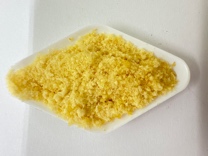 |
| 58 | Peach | powder | *Prunus persica* | NATUREX, Avignon, France |  | 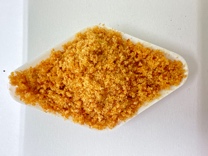 |
| 59 | Apricot | powder | *Prunus armeniaca.* | NATUREX, Avignon, France |  | 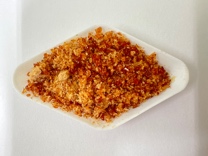 |
| 60 | Mango | powder | *Mangifera indica* | NATUREX, Avignon, France |  | 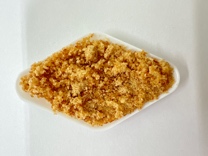 |

^1^ as declared by producer
