# Supplementary figures and images for: Antioxidant Potential of a Wide Range of Commercial Fruit Powders and Grits for Food Applications
Source: Int J Food Sci. 2026 Jun 10;2026:8843447. doi: 10.1155/ijfo/8843447 (PMC13250768; doi:10.1155/ijfo/8843447)

**Table S1b. The photography of black, red, and other dried fruit**

| **No.** | **Photography** |
| --- | --- |
| 1 | 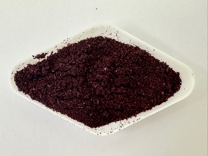 |
| 2 | 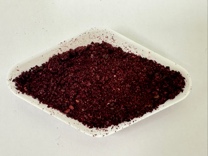 |
| 3 | 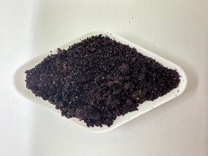 |
| 4 | 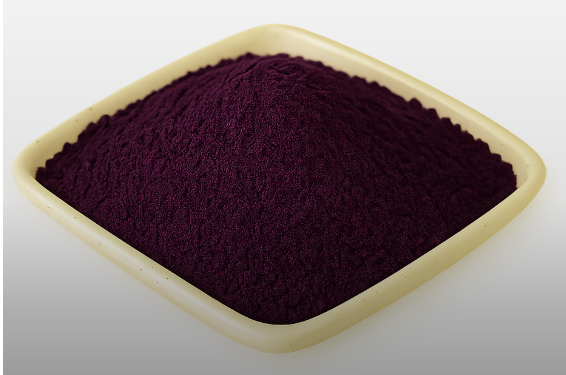 |
| 5 | 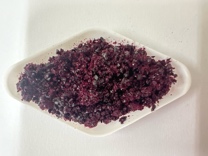 |
| 6 | 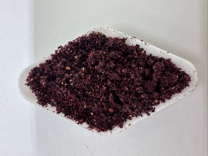 |
| 7 | 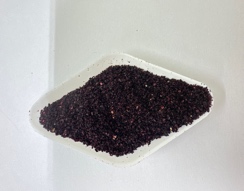 |
| 8 | 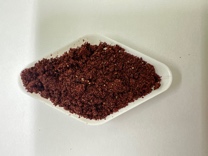 |
| 9 | 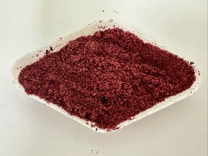 |
| 10 | 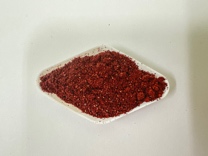 |
| 11 | 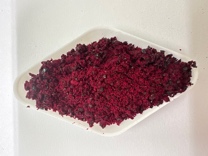 |
| 12 | 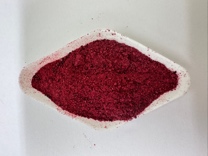 |
| 13 | 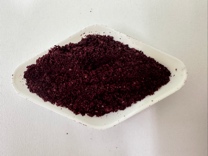 |
| 14 | 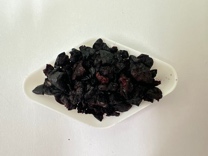 |
| 15 | 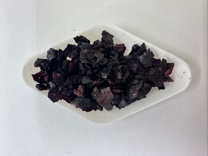 |
| 16 | 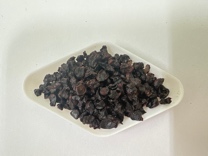 |
| 17 | 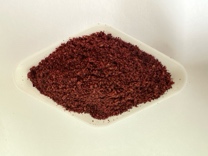 |
| 18 | 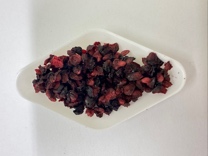 |
| 19 | 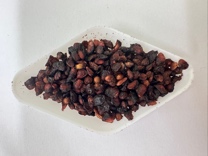 |
| 20 | 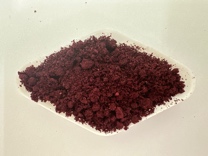 |
| 21 | 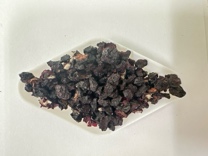 |
| 22 | 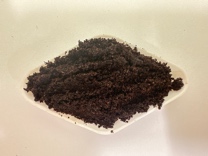 |
| 23 | 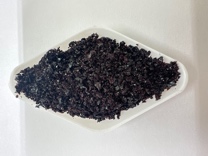 |
| 24 | 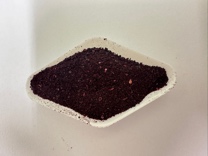 |
| 25 | 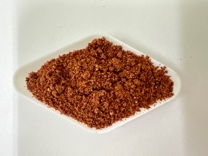 |
| 26 | 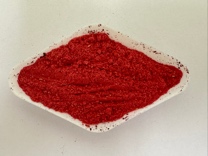 |
| 27 | 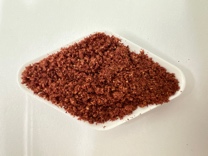 |
| 28 | 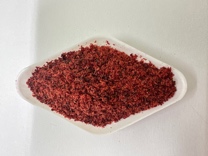 |
| 29 | 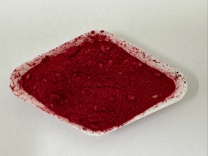 |
| 30 | 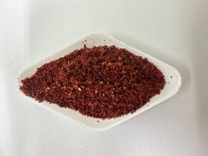 |
| 31 | 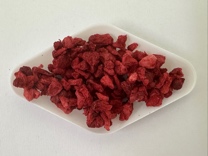 |
| 32 | 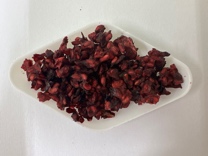 |
| 33 | 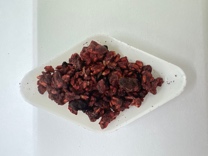 |
| 34 | 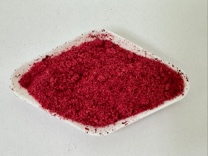 |
| 35 | 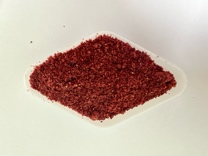 |
| 36 | 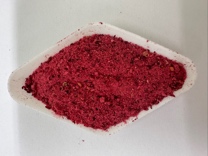 |
| 37 | 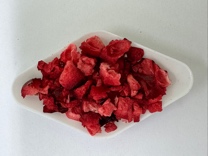 |
| 38 | 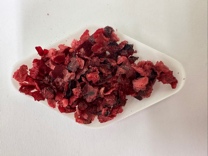 |
| 39 | 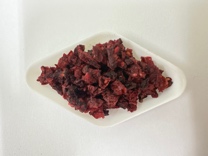 |
| 40 | 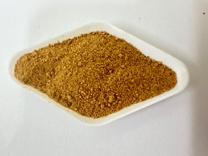 |
| 41 | 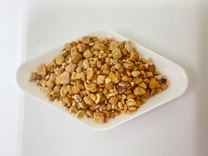 |
| 42 | 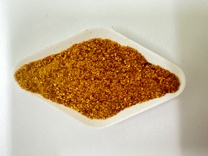 |
| 43 | 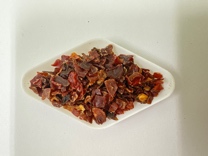 |
| 44 | 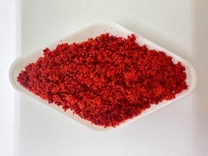 |
| 45 | 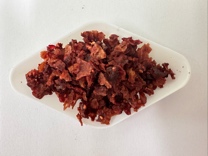 |
| 46 | 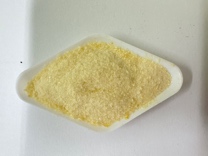 |
| 47 | 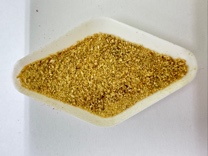 |
| 48 | 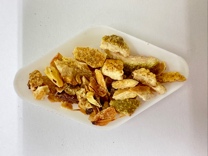 |
| 49 | 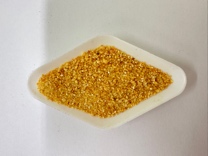 |
| 50 | 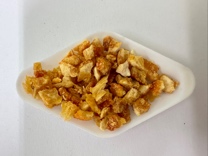 |
| 51 | 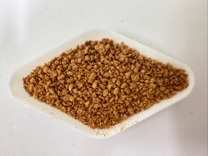 |
| 52 | 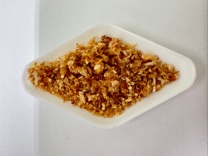 |
| 53 | 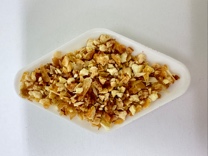 |
| 54 | 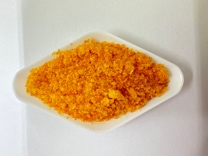 |
| 55 | 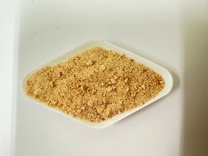 |
| 56 | 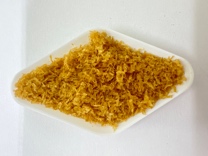 |
| 57 | 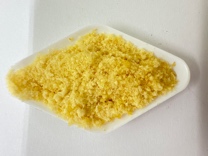 |
| 58 | 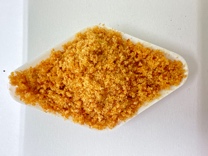 |
| 59 | 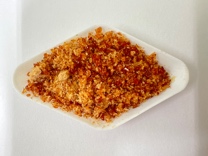 |
| 60 | 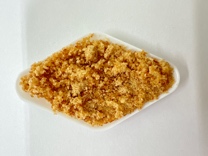 |

Supplement: Supplementary file 1 — Supporting Information Additional supporting information can be found online in the Supporting Information section. Table S1: The characteristics of black, red, and other dried fruit (name, form, Latin name, producer, and additional information). [file IJFO-2026-8843447-s001.zip › Supplementary Material_Additional figures.docx]
